# When glass relaxation is governed by chemical bonding heterogeneity

**DOI:** 10.1093/nsr/nwag175

**Published:** 2026-04-08

**Authors:** En (Evan) Ma

**Affiliations:** Center for Alloy Innovation and Design (CAID), Xi’an Jiaotong University, China

‘Structure determines properties’ is a cornerstone of materials science. For crystalline metals, the periodic arrangement of atoms constitutes a lattice structure, facilitating the establishment of its causal relationship with electronic, mechanical and transport properties. However, this paradigm faces a formidable challenge when crossing the boundary into the disordered world of metallic glasses (MGs) [1–3]. Researchers have long sought to decode hidden order in disorder; they have deciphered the ‘structure’ primarily based on atomic packing motifs, for example the dominant ones that promote the metastability of the glassy state, or the geometrically unfavored ones that tend to respond first to externally imposed stress fields due to their far-from-normal coordination number (CN) [4]. In general, the locally favored structures arise from not only topological short-range order (SRO) to achieve high packing efficiency, but also chemical SRO to maximize preferred bonds and minimize disliked neighbors. The cooperation of these two SRO aspects, together, determines which atomic species and how many of them (CN) form the nearest-neighbor shell making up the coordination polyhedra. Furthermore, the representative motifs must also satisfactorily account for all the constituent elements in their correct molar proportions (i.e. the local chemical make-up should match the alloy composition). Both the geometrical and chemical attributes are expected to influence the flexibility of the local structure [5], when it comes to correlating with the deformability and relaxation dynamics of the glass [4,5].

Writing in *National Science Review* recently, Gao *et al.* conducted a striking case study beyond the common approach above. They singled out a pair of Pd-based metal–metalloid glasses, Pd_40_Cu_40_P_20_ versus Pd_40_Ni_40_P_20_ [6]: these two alloys have the same (i.e. statistically indistinguishable) geometric scheme for atomic packing motifs, but exhibit distinctly different dynamic behavior. This scenario allowed the authors to exclusively attribute the contrasting relaxation property of the MGs to their different bonding environment. Such a demonstration is particularly instructive in highlighting the explicit role of the chemical bonds involved. Previously, the influence of the bonding heterogeneity on the atomic flexibility was obscured by geometrical constraints including volumetric and symmetry effects [5].

**Figure 1.**
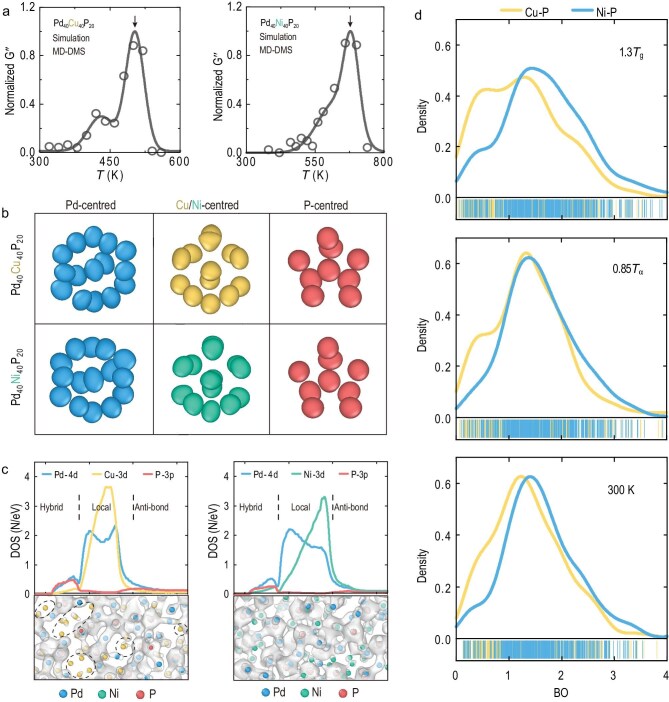
Relaxation dynamics governed by heterogeneity of electronic interactions. (a) Normalized loss spectra *G′* of Pd_40_Cu_40_P_20_ and Pd_40_Ni_40_P_20_ MG. (b) Representative local configurations centered on Pd, Cu/Ni and P atoms. (c) Electronic density of states and charge-density maps. (d) Bond order (BO) distributions for neighboring Cu–P and Ni–P bonds at representative temperatures of 1.3*T*_g_, 0.85*T*_α_ and 300 K (*T*_g_, glass transition temperature; *T*_α_, α-relaxation temperature).

Specifically, the Pd_40_Cu_40_P_20_ and Pd_40_Ni_40_P_20_ MGs are like ‘identical twins’ structurally; comprehensive geometric characterizations—including Voronoi statistics, cluster alignment and smooth overlap of atomic positions (SOAP)-based similarity—indicate that their atomic arrangements are nearly the same (Fig. 1b). Dynamically, however, their relaxation behaviors are strikingly different (Fig. 1a). Pd_40_Cu_40_P_20_ exhibits one of the strongest known secondary (β) relaxations, while its Ni counterpart displays a suppressed β relaxation (manifesting only as an excess wing). As such, the geometrical packing cannot account for their contrast in relaxation dynamics. The authors went on to demonstrate that the decisive parameter is the chemical bonding heterogeneity—spatial fluctuations in interaction strength rooted in electronic structure.

To this end, the authors employed cutting-edge deep-learning molecular dynamics. By training deep neural networks on extensive density functional theory (DFT) data, they achieved *ab initio* accuracy with the scale capable of capturing long-time relaxation processes. This enabled them to quantitatively reproduce the experimental dynamic mechanical spectra *in silico—*a feat rarely achieved in glass physics (Fig. 1a), making it possible to map macroscopic relaxations onto specific microscopic motion patterns.

The study reveals that the secret of relaxation contrast lies not only in where the atoms are, but also in how they are bonded (Fig. 1c). Electronic structure analysis unveils the following fundamental difference. In Pd_40_Cu_40_P_20_, Cu participates in many comparatively weak Cu–P links, which act as ‘soft connectors’ that allow cage breaking and string-like cooperative displacements; the resulting mobile domains can connect and percolate, providing an efficient pathway for a prominent β relaxation. In Pd_40_Ni_40_P_20_, by contrast, stronger Ni–P interactions build a more uniform, interconnected ‘covalent-like’ network. The development of a pseudo-gap during cooling signals rapid electronic stabilization, which stiffens the bonding landscape, suppresses mobility, weakens the β process, and simultaneously enhances resistance to crystallization to give rise to higher glass-forming ability.

Further bond-order analysis ties these findings to direct, quantifiable indicators (Fig. 1d); Cu–P bonds exhibit a distinct shift toward lower bond strengths relative to Ni–P, consistent with a larger population of weaker bonds. These correlate with enhanced atomic mobility, re-framing β relaxation as a consequence not of packing ‘defects’ or extra volume alone, but instead, of spatially variable bond strengths that open low-energy-cost rearrangement pathways to impart extra flexibility volume [4,5] for easier glass reconfiguration.

In a nutshell, beyond ‘how atoms are arranged’, an explicit answer is required as to ‘how strongly—and how heterogeneously—they are bonded’. This insight not only resolves the specific mystery of Pd-based glasses but also points to a general strategy for engineering the ‘electronic interaction landscape’ to tailor the mechanical and relaxation properties of amorphous materials.
